# Complement Activation by an Anti-Dengue/Zika Antibody with Impaired Fcγ Receptor Binding Provides Strong Efficacy and Abrogates Risk of Antibody-Dependent Enhancement

**DOI:** 10.3390/antib12020036

**Published:** 2023-05-15

**Authors:** Zenjiro Sampei, Christine Xing’er Koo, Frannie Jiuyi Teo, Ying Xiu Toh, Taku Fukuzawa, Siok Wan Gan, Takeru Nambu, Adrian Ho, Kiyofumi Honda, Tomoyuki Igawa, Fariyal Ahmed, Cheng-I Wang, Katja Fink, Junichi Nezu

**Affiliations:** 1 Chugai Pharmaceutical Co., Ltd., Yokohama 244-8602, Japan; 2 Chugai Pharmabody Research Pte. Ltd., Singapore 138623, Singapore; 3 Singapore Immunology Network, Agency for Science, Technology and Research, Singapore 138648, Singapore

**Keywords:** antibody engineering, Fc engineering, anti-virus antibody, complement, C1q, Fcγ receptor, antibody-dependent enhancement, dengue, Zika

## Abstract

To combat infectious diseases, vaccines are considered the best prophylactic strategy for a wide range of the population, but even when vaccines are effective, the administration of therapeutic antibodies against viruses could provide further treatment options, particularly for vulnerable groups whose immunity against the viruses is compromised. Therapeutic antibodies against dengue are ideally engineered to abrogate binding to Fcγ receptors (FcγRs), which can induce antibody-dependent enhancement (ADE). However, the Fc effector functions of neutralizing antibodies against SARS-CoV-2 have recently been reported to improve post-exposure therapy, while they are dispensable when administered as prophylaxis. Hence, in this report, we investigated the influence of Fc engineering on anti-virus efficacy using the anti-dengue/Zika human antibody SIgN-3C and found it affected the viremia clearance efficacy against dengue in a mouse model. Furthermore, we demonstrated that complement activation through antibody binding to C1q could play a role in anti-dengue efficacy. We also generated a novel Fc variant, which displayed the ability for complement activation but showed very low FcγR binding and an undetectable level of the risk of ADE in a cell-based assay. This Fc engineering approach could make effective and safe anti-virus antibodies against dengue, Zika and other viruses.

## 1. Introduction

Dengue is a systemic viral infection transmitted between humans by mosquitoes. Dengue virus (DENV) infection in humans is often inapparent but can lead to a wide range of clinical manifestations, from mild fever to potentially fatal dengue shock syndrome [1,2]. There are four distinct dengue serotypes, termed DENV-1, DENV-2, DENV-3, and DENV-4, and infection with any of the four virus serotypes can result in lifelong immunity to that specific serotype. However, a sub-optimal concentration of anti-DENV antibodies during a secondary infection with a different serotype may result in a more severe form of dengue because of a phenomenon known as antibody-dependent enhancement (ADE) [3,4]. Several studies have identified Fcγ receptors (FcγRs) as the critical mediators of ADE in dengue pathogenesis, which can support FcγR-mediated cellular uptake of the virus-antibody immune complexes and subsequent infection and viral production [4,5,6].

Developing an effective dengue vaccine has been challenging partly because of the need to protect against all four DENV serotypes simultaneously [7,8]. Furthermore, because an insufficient level of neutralizing antibodies may cause ADE of DENV infection, the vaccination must induce the production of a sufficient number of effective antibodies. Another potential concern about vaccination is the insufficient cross-reactivity of produced antibodies toward other flaviviruses, including the Zika virus (ZIKV), which can be linked to the ADE of flavivirus infections [9]. There is currently one FDA-approved dengue vaccine, CYD-TDV (Dengvaxia^®^), which has some limitations in its clinical use and second-generation vaccines that have displayed improved safety and protection rates in clinical trials [8,10]. However, even if these vaccines are successfully developed, further treatment options are needed for vulnerable populations whose immunity against dengue is immature or who have a high risk of ADE.

Therapeutic antibodies that enhance viral clearance can potentially prevent severe dengue and aid in faster recovery from the disease symptoms. Moreover, antibody engineering can suppress the potential risk of ADE, which could be caused by these antibodies [11]. SIgN-3C, a human antibody that exerts cross–neutralizing activity against all four DENV serotypes by recognizing their envelope (E) proteins, was isolated from a patient infected with DENV [12]. The antibody also shows neutralizing capacity against ZIKV [13], whose E protein has high sequence similarity with that of DENV [14]. And interestingly, the antibody inhibits DENV fusion with host cells, while it aggregates ZIKV [15]. To minimize the risk of ADE, the L234A/L235A (LALA) substitution [16] was introduced, and the SIgN-3C-LALA antibody showed efficacy in both prophylactic and therapeutic settings, together with the suppressed ADE risk in vitro [12]. To improve the clinical utility of this antibody, we implemented protein engineering, such as affinity maturation toward E proteins of the four serotypes and optimization of the Fc function. The engineered antibody, termed AID351, is currently being developed.

It has been recently demonstrated that intact Fc effector functions are required for optimal therapeutic protection by some neutralizing antibodies against SARS-CoV-2, while they are dispensable when administered as prophylaxis [17,18]. Although abrogation of antibody binding to FcγRs is preferred when considering the ADE risk of DENV or ZIKV, such Fc engineering might affect the protective efficacy. Hence, in this report, we investigated the influence of Fc engineering on anti-DENV efficacy using the SIgN-3C antibody. Our results suggest that eliminating Fc effector functions might affect the viremia clearance activity of the antibody, in which complement activation is likely involved. We also identified a potential Fc engineering approach for anti-virus antibodies against dengue, Zika, and possibly other viruses.

## 2. Materials and Methods

### 2.1. Preparation of Proteins and Antibodies

The genes encoding the extracellular region of human, and mouse FcγRs were synthesized based on the sequence information obtained from the National Center for Biotechnology Information. FcγRs were fused with 6× His-tag at their C-terminus. The FcγRs were transiently expressed using the FreeStyle 293 Expression System (Thermo Fisher Scientific, Waltham, MA, USA) and purified from the harvested culture supernatants by ion exchange chromatography, nickel affinity chromatography, and size exclusion chromatography. Recombinant antibodies were transiently expressed using the FreeStyle 293 Expression System or Expi293 Expression System (Thermo Fisher Scientific). Purification was performed with a conventional method using protein A. Gel filtration was conducted when necessary.

### 2.2. Evaluation of Binding Activity to FcγRs

The interaction of Fc variants with human or mouse FcγRs was measured using a Biacore T200 instrument (GE Healthcare, Uppsala, Sweden). Although forming large ICs can improve the detection of the binding activity to FcγRs, a combination of anti-DENV antibodies and soluble E-proteins cannot form large ICs. To better reflect the virus-antibody complexes, we identified an anti-CD154 antibody that could form large ICs in the presence of trimeric CD154 (prepared in-house). The large CD154-antibody ICs, which can be evaluated by size-exclusion chromatography analysis, exhibit avidity binding to the FcγRs. Each antibody variant and CD154 were mixed with a 1:1 molar ratio and incubated at room temperature for 1 h to form ICs. FcγRs were captured on a Sensor Chip CM4 (GE Healthcare) on which anti-histidine antibody (GE Healthcare) was immobilized, followed by injection of antibody alone or the mixture of antibody and CD154. The binding activity was determined based on the binding response, normalized by the capture level of each FcγR.

### 2.3. Evaluation of Binding Activity to Human C1q

The interaction of Fc variants with human C1q was measured by ELISA. MaxiSorp 384-well plates (Thermo Fisher Scientific) were directly coated with the anti-CD154 antibodies overnight at 4 °C. After blocking with TBS-T containing 0.5% BSA and 1× Block Ace (DS Pharma Biomedical, Osaka, Japan) for 2 h at room temperature, 3 μg/mL of human C1q (Sigma-Aldrich, St. Louis, MO, USA) was added onto the plates and incubated for 1 h at room temperature. At room temperature, the plates were incubated with sheep anti-human C1q antibody-HRP conjugate (Bio-Rad, Hercules, CA, USA) for 1 h. Washes in PBS-T (pH 7.4) were performed after each subsequent step. TMB substrate (Thermo Fisher Scientific) was subsequently added, and the signal was measured by a plate reader at a wavelength of 450 nm (test wavelength) and 570 nm (reference wavelength).

### 2.4. Evaluation of Binding Activity to Mouse C1q

The interaction of Fc variants with mouse C1q was measured by ELISA. MaxiSorp 384-well plates (Thermo Fisher Scientific) were directly coated with the anti-CD154 antibodies overnight at 4 °C. After blocking with TBS-T containing 0.5% BSA and 1 × Block Ace (DS Pharma Biomedical) for 7 h at 4 °C, 10% mouse plasma (Innovative Research, Novi, MI, USA) was added onto the plates and incubated overnight at 4 °C. At room temperature, the plates were incubated with a biotinylated anti-mouse C1q antibody (Hycult Biotech, Uden, The Netherlands) for 1 h. Pierce High Sensitivity Streptavidin-HRP (Thermo Fisher Scientific) was added to react for 1 h at room temperature. Washes in PBS-T (pH 7.4) were performed after each subsequent step. ABTS peroxidase substrate (SeraCare Life Sciences, Milford, MA, USA) was subsequently added, and the signal was measured by a plate reader at a wavelength of 405 nm.

### 2.5. In Vivo Efficacy Study

Six- to 13-week-old AG129 mice were infected intraperitoneally with 10^6^ plaque-forming units (pfu) of DENV-2 strain D2Y98P. Forty-eight hours later, the mice were treated with 1–30 μg of the antibody, which was injected intravenously via the retro-orbital route. A further 24 h later, that is, 72 h after the initial infection, blood was collected. It has been established that peak viremia after infection with D2Y98P is reached between days 3–4 after infection in previous studies [19,20]. Viral RNA was extracted from the plasma of each mouse, and a quantitative PCR was performed and compared against a DENV-2 standard with known infectivity in a plaque assay. Body weight was monitored twice a week, beginning on the day of infection until the experiment’s endpoint. Mice that showed 20% body weight loss for more than 48 h and/or moribund mice were euthanized. Euthanized mice were recorded as dead for survival analysis.

### 2.6. In Vitro Complement Activation Assay

The ability of complement activation by the Fc variants was measured by ELISA. MaxiSorp 384-well plates (Thermo Fisher Scientific) were directly coated with the antibodies for 1 h at room temperature. After blocking with TBS-T containing 0.5% BSA and 1× Block Ace (DS Pharma Biomedical) for 2 h at room temperature, pooled human complement serum (Innovative Research) or AG129 mouse serum was added onto the plates and incubated for 1 h at 25 °C. The plates were incubated with anti-C3b antibody clone 6c9 (Thermo Fisher Scientific) for 1 h at 25 °C, followed by incubation with a peroxidase-conjugated antibody to mouse IgG_1_ (Yamasa Corporation, Chiba, Japan) for 30 min at room temperature. Washes in PBS-T (pH 7.4) were performed after each subsequent step. TMB substrate (Thermo Fisher Scientific) was subsequently added, and the signal was measured by a plate reader at a wavelength of 450 nm. The complement activation was confirmed to be suppressed in this assay when adding EDTA into the serum.

### 2.7. In Vitro ADE Assay

Dengue virus seed stocks were diluted to achieve a multiplicity of infection (MOI) of 1, which were mixed with various concentrations of the antibodies in either non-heat-inactivated serum or heat-inactivated serum and incubated at 37 °C for 1.5 h. The virus-antibody mixture was added to K562 (ATCC, Manassas, VA, USA) cells and incubated at 37 °C for 1.5 h. After incubation, complete media were added to cells and incubated at 37 °C for 2.5 days. Infection was assessed by flow cytometry on a FACSVerseTM Cell Analyzer (BD Biosciences, San Jose, CA, USA) using antibodies for E (Ab 4G2, hybridoma purchased from ATCC) and NS1 protein that were stained intracellularly.

## 3. Results

### 3.1. Therapeutic Efficacy in Mice Was Affected by Fc Engineering

**Figure 1:**
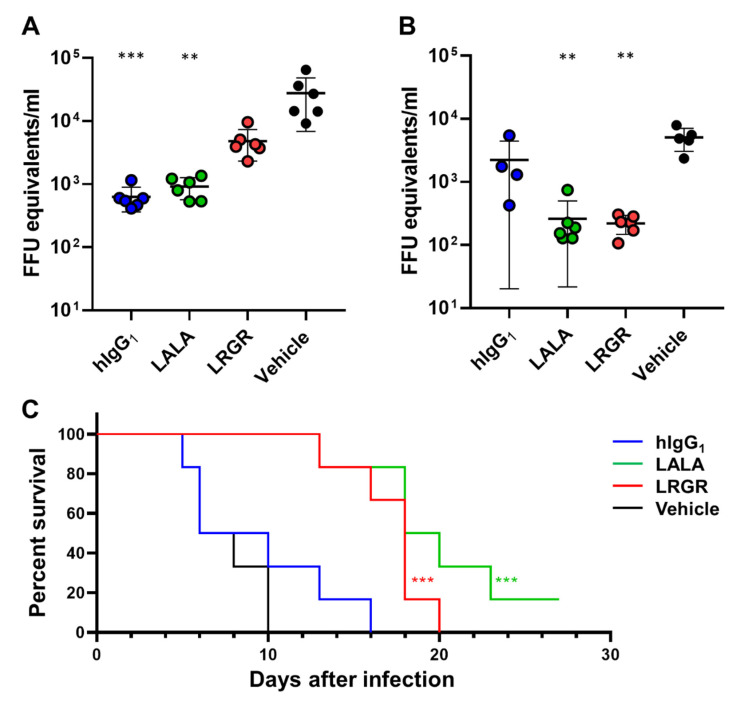
Effect of Fc engineering on in vivo efficacy of anti-DENV antibody. AG129 mice were challenged with DENV-2, and 10 μg of the antibody, hIgG_1_ (blue), LALA (green), or LRGR (red) was administered two days after the virus challenge. The viremia was measured on day three (**A**) and day five (**B**) after the antibody injection. Each symbol represents one mouse and means ± SD are shown. Asterisks indicate statistical differences compared to a vehicle control group with Dunn’s multiple comparison test for the viremia on days three and five (**, *p* < 0.01; ***, *p* < 0.001). Survival analysis was conducted in this study (**C**). Asterisks indicate statistical differences compared to the vehicle control group with the Mantel-Cox test for the survival analysis (***, *p* < 0.001).

To investigate the potential effects of Fc engineering on efficacy, we prepared SIgN-3C antibody derivatives with substitutions in the Fc region, L234A/L235A (LALA) [16] or L235R/G236R (LRGR). LRGR has been used for our antibodies [21] because it suppresses FcγR binding more strongly than the LALA substitution. We administered 10 μg of the antibody with the following Fc regions, human IgG_1_ (hIgG_1_), LALA, or LRGR, two days after AG129 mice were challenged with DENV-2 (therapeutic model). The viremia was determined on days three and five after the antibody injection, and a survival analysis was performed (Figure 1). While the hIgG_1_ and LALA antibodies showed significant in vivo efficacy at day 3, the LRGR antibody did not strongly suppress the viremia (Figure 1A).

**Figure 2:**
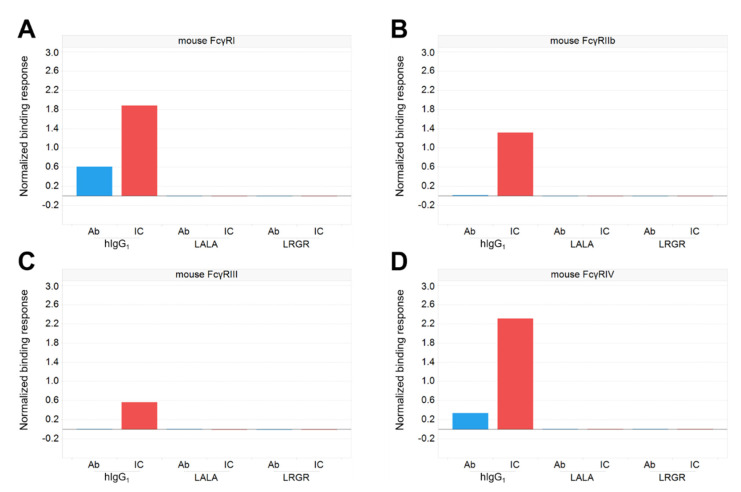
Binding activity of Fc-engineered antibodies to mouse FcγRs. Anti-CD154 antibodies with the Fc variants hIgG_1_, LALA, or LRGR were prepared, and immune complexes (ICs) were formed by adding CD154 to allow strong avidity binding to FcγRs. The binding of the antigen-free antibodies (blue) and ICs (red) of each antibody to mouse FcγRI (**A**), FcγRIIb (**B**), FcγRIII (**C**), and FcγRIV (**D**) were measured by SPR analysis.

To investigate whether FcγR binding played a role in the mechanism of the weakened early efficacy of the LRGR antibody, we measured the binding activity of the Fc variants to mouse FcγRs by using an anti-CD154 antibody as a model. The mouse FcγR binding was suppressed by either LALA or LRGR, even when the antibodies formed large immune complexes (ICs) by incorporating a multimer antigen CD154 (Figure 2). However, only the LRGR variant of SIgN-3C showed weaker in vivo efficacy (Figure 1A), suggesting that LRGR affected another factor involved in viremia reduction efficacy that was not directly related to the FcγR-mediated mechanism.

### 3.2. Antibody Engineering for Therapeutic Application

**Figure 3:**
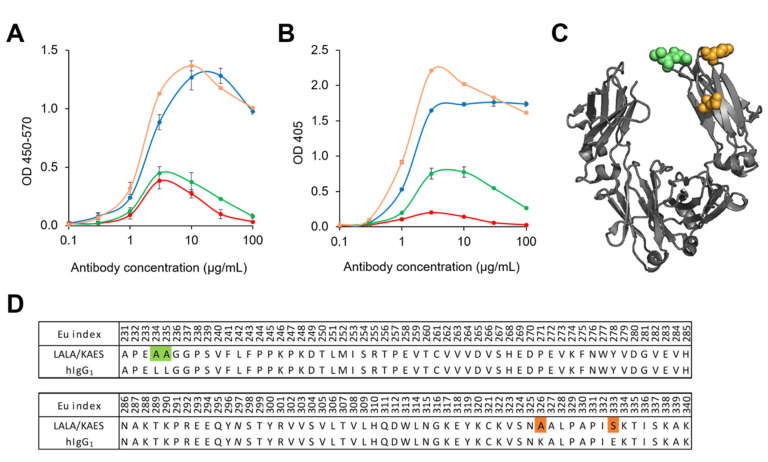
Binding activity of Fc-engineered antibodies to human/mouse C1q. The binding activity of the anti-CD154 antibody, hIgG_1_ (blue), LALA (green), LRGR (red), or LALA/KAES (orange) to human C1q (**A**) or mouse C1q (**B**) was measured. The positions of the substitutions, LALA (green) and KAES (orange), are shown in the illustration of the hIgG_1_ Fc region (PDBID:3AVE) (**C**) and the sequence alignment (**D**). Figure 1C was generated with PyMOL.

Since the complement system has been reported to play an important role in flavivirus infection [22], we next measured the binding activity of the Fc variants to human and mouse C1q (Figure 3). While LALA and LRGR reduced human C1q binding to a similar level (Figure 3A), mouse C1q binding was reduced more strongly by LRGR than LALA (Figure 3B). This strong reduction in mouse C1q binding by LRGR could explain the impaired efficacy. To minimize the risk of impaired efficacy in humans, we screened substitutions restoring the C1q binding reduced by the LALA substitution and found the adequate substitution K326A/E333S (KAES). The LALA/KAES Fc variant exhibited binding to human and mouse C1q comparable to or even stronger than hIgG_1_.

### 3.3. In Vivo Efficacy Was Improved by Fc Engineering to Enhance C1q Binding

**Figure 4:**
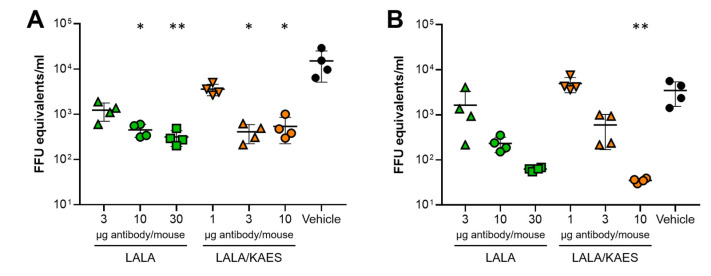
In vivo efficacy of Fc-engineered antibodies. AG129 mice were challenged with DENV-2, and the antibody, LALA (green) or LALA/KAES (orange), was administered two days after the virus challenge. The viremia was determined on day three (**A**) and day five (**B**) after the antibody injection. The variable region of the anti-DENV antibodies used in this experiment was an engineered variant termed 3Cam2, which has improved affinity toward E-proteins of the four DENV serotypes. Each symbol represents one mouse and means ± SD are shown. Asterisks indicate statistical differences compared to the vehicle control group with Dunn’s multiple comparison test for the viremia on days three and five (*, *p* < 0.05; **, *p* < 0.01).

We conducted an in vivo efficacy study in mice, in which we compared LALA and LALA/KAES (Figure 4). To achieve a similar level of viremia reduction, the amount of the LALA/KAES antibody was smaller than that of the LALA antibody. Comparing the difference from the vehicle group, 3 μg of LALA/KAES showed significance, but 3 μg of LALA did not at day 3 (Figure 4A), and 10 μg of LALA/KAES showed significance but 10 μg or 30 μg of LALA did not at day 5 (Figure 4B). These results suggest that enhanced C1q binding could improve the in vivo efficacy.

**Figure 5:**
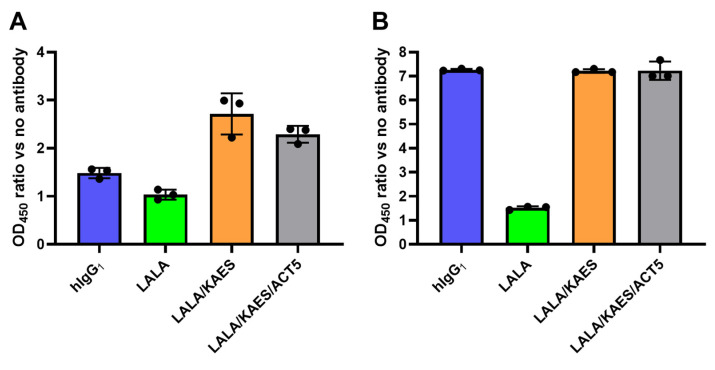
Complement activation by Fc-engineered antibodies. The antibodies, human IgG_1_ (blue), LALA (green), LALA/KAES (orange), and LALA/KAES/ACT5 (gray), were directly immobilized on the assay plate and incubated with human (**A**) or AG129 mouse (**B**) serum. The increased amount of C3b was measured as an indicator of complement activation. The vertical axes represent the absorbance ratio versus the well without antibody immobilization. The variable region of the anti-DENV antibodies used in this experiment was an engineered variant termed 3Cam2, which has improved affinity toward E-proteins of the four DENV serotypes.

We also compared the ability of complement activation by the Fc variants. The antibodies were immobilized on the assay plate and incubated with human or mouse serum, after which the C3b level was measured as an indicator of complement activation. Wild-type human IgG_1_ induced C3b, which was weakened by LALA but was recovered by KAES in human and mouse serum (Figure 5A,B). The Fc engineering to enhance FcRn binding (ACT5) [23], which prolongs the pharmacokinetics of the antibody, did not affect the complement activation activity in this assay.

### 3.4. No ADE Risk Was Observed by the Engineered Antibody AID351 In Vitro

**Figure 6:**
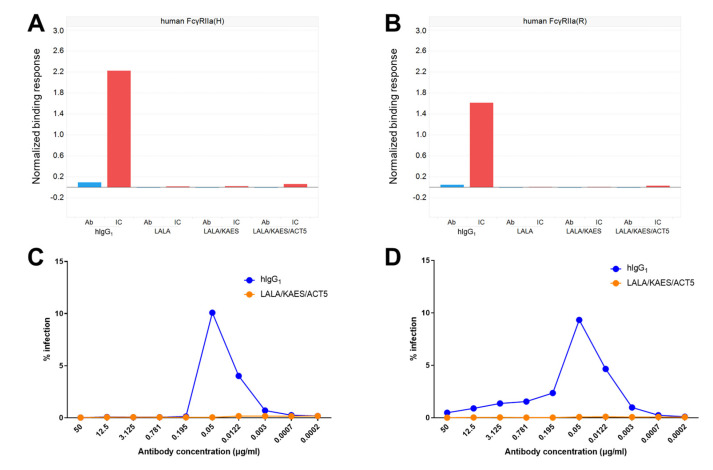
FcγR binding and ADE risk of Fc-engineered antibodies. Anti-CD154 antibodies with the Fc variants, hIgG_1_, LALA, LALA/KAES, or LALA/KAES/ACT5, were prepared and immune complexes (ICs) were formed by adding CD154 to allow strong avidity binding to FcγRs. The binding of the antigen-free antibodies (blue) and ICs (red) of each antibody to human FcγRIIa-H131 (**A**) and human FcγRIIa-R131 (**B**) was measured by SPR analysis. In addition, the potential ADE risk of the anti-DENV antibodies on K562 cells against DENV-2 in non-heat-inactivated serum (**C**) or in heat-inactivated serum (**D**) was assessed by quantifying the virus in the supernatant of K562 cells. Enhancement observed with the human IgG_1_ (blue) was abrogated with the LALA/KAES/ACT5 variant (orange). The variable region of the anti-DENV antibodies was an engineered variant termed 3Cam2, which has improved affinity toward E-proteins of the four DENV serotypes.

Among human FcγRs, FcγRIIa is known to be the main mediator of ADE [24]. To evaluate the potential effect of the Fc engineering on ADE, we measured the binding activity of the Fc variants to two allelic forms of human FcγRIIa (H131 and R131) using an anti-CD154 antibody (Figure 6A,B). Compared with human IgG_1_, the other Fc variants with the LALA substitution showed much weaker or undetectable binding levels to human FcγRIIa, even when antibodies formed large ICs by incorporating a multimer antigen CD154. We then examined the potential ADE risk of the affinity matured (3Cam2) and Fc-engineered (LALA/KAES/ACT5) antibody variant by using an in vitro assay consisting of dengue-infected FcγRIIa-expressing K562 cells. We used the non-heat-inactivated or heat-inactivated serum in this assay, and no ADE risk was observed regardless of the presence of complement activity (Figure 6C,D).

## 4. Discussion

Although vaccines are considered the best prophylactic strategy for a wide range of the population, several therapeutic antibodies for preventing or treating viral infections are in development [25,26]. The current COVID-19 pandemic demands a variety of prophylactic and therapeutic options to combat the viruses, and therapeutic antibodies are potentially beneficial for patients as they can reduce the risk of hospitalization or death [27,28,29,30]. Similarly, against dengue, a neutralizing antibody could be effective as a medical intervention, particularly when vaccination does not induce antibodies with sufficient neutralizing activity against any of the four DENV serotypes. We, therefore, engineered the SIgN-3C antibody, which showed broadly neutralizing activity against all four DENV serotypes and ZIKV [12,13], as a potential pharmaceutical product.

Antibody engineering has been applied to several therapeutic antibodies as it can improve efficacy, safety, and convenience for patients and caregivers. Among the variable region engineering approaches [31], affinity maturation of a neutralizing antibody toward its target antigen is a promising way to improve its efficacy. However, there are four serotypes of DENV to be neutralized, each of which has a different sequence in its envelope protein. Affinity maturation toward the E-protein of a specific serotype could reduce the potency against other dengue serotypes or ZIKV, which may lead to the lack of efficacy against the other serotypes. Although the SIgN-3C antibody showed neutralizing activity against all four serotypes, the efficacy against the DENV-3 serotype appeared weaker than the others. We performed comprehensive substitution [21] and generated affinity-improved antibody variants by carefully considering the effect on the binding activity to all four serotypes. We then generated 3Cam2, which showed improved binding activity against all four serotypes. Sub-neutralizing antibodies with active FcγR binding produced in some patients who have been previously infected or vaccinated are known to cause ADE. Therapeutic antibodies can minimize the risk by competing with endogenous antibodies. Thus, such therapeutic antibodies with higher affinity will also be safer in patients.

Fc engineering to modify the binding activity to FcγRs, C1q, or FcRn has also been well studied [32], and selectively enhanced binding to specific FcγRs has been achieved [33,34,35]. Since ADE is a potential safety concern for vaccines or antibody drugs against dengue, suppressing the FcγR binding of anti-DENV therapeutic antibodies is preferred. Although SIgN-3C-LALA was demonstrated to show efficacy in both the prophylactic and therapeutic settings [12], some neutralizing antibodies against SARS-CoV-2 required intact Fc effector functions for optimal therapeutic protection [17]. Similarly, for anti-DENV antibodies, strongly suppressing FcγR binding by LRGR resulted in impaired viremia reduction in the therapeutic setting (Figure 1A). The result on day 5 suggests that LRGR can be beneficial to a certain degree, and hIgG_1_ has the risk of weakened efficacy because of ADE (Figure 1B), which motivated us to engineer the Fc function for further improvement. Comparing LALA and LRGR, we observed a difference in the binding capacity to mouse C1q, which may have contributed to the difference in viremia reduction [22,36].

C1q has been shown to inhibit ADE for DENV and West Nile virus (WNV) and to improve the antibody-mediated neutralization of WNV in vitro [37] and in vivo [38]. Furthermore, it was proposed that C1q decreased the number of antibodies required per virus particle to achieve neutralization [37]. We thus assumed that enhancing the C1q binding of anti-DENV antibodies would exhibit improved efficacy. The C1q binding site of human IgG_1_ has been investigated [39], and K326W/E333S enhanced the C1q binding capacity and CDC activity of rituximab [40]. In addition to the engineering at positions K326 and E333, several engineered Fc variants have also been reported to exert enhanced CDC activity [41,42,43]. Considering the safety risk of FcγR-mediated ADE, therapeutic antibodies against dengue are preferred to have engineered Fc with abolished FcγR binding and active C1q binding capacity. Although some amino acid substitutions that can suppress ADCC but maintain CDC activity have been reported [40,44], effects on other complement-mediated bioactivity or ADE have not been investigated. We, therefore, evaluated the C1q and FcγR binding activity of some of the substitutions reported [40,41]. However, because none of the substitutions we tested showed sufficient C1q binding with completely suppressed FcγR binding for ICs, they were unsuitable for anti-dengue antibody drugs due to the risk of ADE. To identify a novel Fc variant for improved efficacy, safety, and developable pharmaceutical properties, we generated and evaluated several Fc variants and selected LALA/KAES as a candidate. K326 and E333 are considered to play a structural role in interaction with C1q [40] and are in close proximity to L234 and L235, which are engineered to suppress FcγR binding (Figure 3C). Therefore, LALA and KAES are likely to interfere with each other, and the combination ended up exhibiting negligible FcγR binding activity with comparable or stronger C1q binding than wild-type human IgG_1_.

We found that the C1q binding-restoring Fc engineering, KAES, positively affected in vivo efficacy in mice (Figure 4). In vitro C1q ELISA data showed that mouse C1q binding was more affected by LRGR than LALA, whereas the binding to human C1q was similar for both substitutions (Figure 3). These results suggest that C1q binding in mice contributed to virus control, as observed in our study (Figure 4), whereas the role of C1q in humans remains unknown. The complement activation study showed a small signal of complement activation by the LALA antibody in mouse serum (~1.5-fold increase versus the well without antibody immobilization). In contrast, no increase was detected in human serum (Figure 5). The results of C1q ELISA and the complement activation assay indicate that the LALA antibody has C1q binding capacity in mice to a certain extent, which could contribute to the viremia reduction in mice. Still, the efficacy of the LALA antibody might be weakened in infected patients, similar to the weakened early efficacy of the LRGR antibody in Figure 1A.

The FcγR binding of the LALA/KAES antibody was strongly suppressed, and KAES did not negatively affect the ADE-abrogating ability of the LALA substitution when using the human cell line K562 as a model ADE system (Figure 6). Therefore, we hypothesize that increased C1q binding could support the neutralization of dengue in humans by a therapeutic or prophylactic antibody and could also improve the safety profile by further suppressing ADE.

Prophylactic usage of antibodies against malaria has been discussed [45,46]. Similarly, our anti-DENV antibody can be administered as prophylaxis for high-risk populations or travelers in endemic regions and its plasma half-life is preferred to be longer. Even if the antibody is injected after DENV infection, long-lasting efficacy could benefit recipients by protecting them from subsequent infection. Therefore, in addition to the Fc engineering to modulate FcγR binding and C1q binding, the FcRn binding of therapeutic antibodies has been engineered to improve the pharmacokinetics [23]. In this study, we examined the combination of the Fc engineering technologies (LALA, KAES, ACT5) and evaluated the pharmaceutical characteristics, including pharmacokinetics. The engineered antibody (affinity maturation and Fc engineering, AID351) showed a long plasma half-life and good subcutaneous and intramuscular bioavailability in cynomolgus monkeys. The good pharmacokinetics of AID351, improved by ACT5, will provide significant benefits in the prophylactic use against dengue and in suppressing ADE risk caused by re-infection.

In this study, abrogating the FcγR binding of an anti-DENV neutralizing antibody impaired antiviral efficacy in a therapeutic model. We assumed that complement activation might play an important role in virus clearance. We successfully identified a novel engineered Fc with negligible FcγR binding but active C1q binding comparable to human IgG_1_. This engineered Fc (LALA/KAES) displayed improved viremia reduction efficacy in mice compared to the LALA antibody. The ADE risk of the antibody was not observed in the in vitro assessment. Since the role of complements and the molecular mechanism underlying viremia reduction by the engineered antibody remain unclear, further experiments are preferred in the future. Although this type of Fc engineering has not been applied for therapeutic usage, it can be considered in antibody drug discovery when complement activation is preferred, but FcγR binding must be suppressed.

## Data Availability

Data is contained within the article.
